# Golimumab Ameliorates Pancreatic Inflammatory Response in the Cerulein-Induced Acute Pancreatitis in Rats

**DOI:** 10.5152/tjg.2022.21456

**Published:** 2022-11-01

**Authors:** Mustafa Kaplan, Alpaslan Tanoğlu, Başak Çakır Güney, Murat Yeniçeri, Zafer Çırak, Yeşim Önal Taştan, Ayşe Gökçen Sade

**Affiliations:** 1 Department of Internal Medicine, Sultan 2. Abdulhamid Han Training and Research Hospital, İstanbul, Turkey; 2 Department of Gastroenterology, Sultan 2. Abdulhamid Han Training and Research Hospital, İstanbul, Turkey; 3 Department of Pathology, Sultan 2. Abdulhamid Han Training and Research Hospital, İstanbul, Turkey

**Keywords:** Acute pancreatitis, golimumab, inflammation, rat

## Abstract

**Background:**

The aim of the study was to evaluate whether a new and successful treatment opportunity can be provided in acute pancreatitis and may prevent symptomatic treatments and show its effect through etiopathogenesis. Therefore, we want to investigate the efficacy of golimumab in an experimental rat model of cerulein-induced acute pancreatitis.

**Methods:**

A total of 35 rats, including 7 rats in each group, were distributed into 5 groups (sham, acute pancreatitis, placebo, acute pancreatitis + golimumab 5 mg/kg, and acute pancreatitis + golimumab 10 mg/kg). An experimental cerulein-induced acute pancreatitis model was accomplished by intraperitoneal cerulein injections. After sacrification, rat blood samples were collected for amylase, IL-6, and IL-1beta measurements. Histopathological analysis of the pancreas was performed with Tunel and hematoxylin & eosin staining.

**Results:**

Amylase, IL-6, and IL-1beta levels were found to be increased in the acute pancreatitis group. IL-1beta, amylase, IL-6 levels, and pancreatic inflammation were all significantly decreased in golimumab groups (*P *< .01). Moreover, in both golimumab groups, golimumab treatment significantly reduced apoptosis in pancreatic tissues (*P *< .05). Golimumab treatment was found to significantly reduce edema formation, inflammation, vacuolization, and fat necrosis of pancreatic tissues (*P *< .05).

**Conclusion:**

Firstly in the literature, we investigated the efficacy of golimumab in the experimental acute pancreatitis model. In the light of our findings, it could be suggested that golimumab may be an effective and safe therapeutic option in the treatment of patients with acute pancreatitis.

**Main Points**

Acute pancreatitis (AP) is a disease that can lead to life-threatening consequences.
Despite the development of treatment methods and intensive care conditions, it is a disease with reported mortality rates between 15% and 40% in severe cases.
Regardless of the etiology of pancreatitis, both acute and chronic pancreatitis pathogenesis may be associated with oxidative stress and inflammation.
Our current study showed that golimumab (GLM) is an effective and safe drug for the treatment of AP. Moreover, it can be suggested that GLM will be a favorable therapeutic option for acute AP.

## Introduction

Acute pancreatitis (AP) is an acute, inflammatory, and life-threatening disease of the pancreas.^1^ Although the annual incidence of acute pancreatitis is 34 per 100 000, it is thought to increase gradually.^2^ Despite the development of all treatment methods and intensive care conditions, it is a disease with reported mortality rates between 15% and 40% in severe cases.^3^ The pathophysiology of AP is still under investigation. Although controversial, most researchers believe that AP develops as a result of impaired trypsin activation regulation. It demonstrates that by revealing acinar cell damage by intracellular activation of enzymes, the pancreatic parenchyma is the trigger of further auto-digestion and local inflammatory response.^4^ Enzyme activation causes autosynthesis and inflammation in the pancreatic gland.^5^ After trypsin activation in acinar cells, elastase, phospholipase A2, complement, and kinin pathways are also activated. Additionally, IL-1, IL-6, IL-8, and TNF-alpha are released from neutrophils, macrophages, and lymphocytes.^6^ The pancreas is very sensitive to ischemia; therefore, it plays a crucial role in ensuring adequate blood flow, homeostasis, and preventing inflammatory conditions.^7^ The pathophysiology of AP, both experimentally and clinically, has not been clearly demonstrated. Although complications of lung, kidney, or liver failure frequently occur in moderate-to-severe acute pancreatitis cases, it still remains unclear how it affects all organs in general.^1,2^ While many systemic-acting cytokines play a role in these uncertain mechanisms, especially IL-1 and TNF-a are cytokines that play a major role. In acute pancreatitis, IL-2, IL-6, IL-8, IL-10, and nitric oxide also contribute to this systemic effect mechanism. These cytokines cause worsening by increasing capillary permeability. At this stage, it is thought that preventing the release of these cytokines will help prevent AP and systemic complications.^1-3^

Currently, there is no globally accepted effective treatment method to treat the clinical picture of AP. Due to these limited treatment options, effective treatments that will affect the pathways in the pathogenesis of the disease are urgently needed.^8^

Golimumab (GLM), a human IgG1 kappa monoclonal antibody, binds specifically to the soluble and transmembrane form of TNF-alpha with high affinity and specificity, thus preventing the binding of the target cell TNF-alpha surface antigen, thereby preventing E-selectin adhesion molecules, vascular cell adhesion molecule, and intercellular adhesion molecules of endothelial cells.^9,10^ It neutralizes the effects of TNF alpha by inhibiting its expression.^9^ Golimumab also has a cellular cytoprotective effect. Golimumab has been shown to reduce the level of reactive oxygen species which are closely related to C-reactive protein, TNF-alpha, and IL-1 and IL-6 expressions.^10-12^ There is no study in the literature that examines the effects of TNF-alpha receptor antagonist GLM on the experimental acute AP models.

In this study, it was aimed to examine the effectiveness of GLM, which has anti-ischemic, anti-apoptotic, and anti-inflammatory properties, in an experimental rat AP model. Thus; we aimed to evaluate whether a new and successful treatment opportunity can be provided in AP and it may prevent symptomatic treatments and show its effect through etiopathogenesis.

## Materials and Methods

### Study Design

This experimental rat study was performed in line with the recommendations of the national guidelines for animal care and handling in the laboratory. All study protocols were approved by the Animal Ethics Committee of the University of Health Sciences (March 6, 2019/2019-03/04). The research was completed at the University of Health Sciences Animal Laboratory Center.

Experimental AP was induced through intraperitoneal (IP) administration of cerulein (Sigma, St. Louis, MO, USA) injections. A total of 35 rats, including 7 rats in each group, were equally distributed into 5 groups. Group 1: Sham group (1 mL of saline was administered to rats as IP injections 4 times at 1-hour intervals, no other drugs were used. Sacrification was done 24 hours after the last injection). Group 2: AP group (40 μg/kg cerulein was administered to rats as IP injection (4 times at 1-hour intervals). The rats were sacrificed 24 hours after the last injection). Group 3: AP + GLM 5 mg/kg group: Cerulein injections were done as mentioned above and then 5 mg/kg GLM was given as an IP injection 12 hours after the last cerulein injection. Rats were sacrificed 12 hours after the last GLM injection. Group 4: AP + GLM 10 mg/kg group: Cerulein injections were done as mentioned above and then 10 mg/kg GLM was given as an IP injection 12 hours after the last cerulein injection. Rats were sacrificed 12 hours after the last GLM injection. Group 5: Placebo group: Cerulein injections were done as mentioned above and 1 cc saline will be given IP as placebo 12 hours after the last cerulein injection. Rats in this group will be sacrificed 12 hours after intraperitoneal saline administration.

Twelve hours after GLM administration, rats were given IP injection of ketamine hydrochloride (40 mg/kg) and xylazine (5 mg/kg), and blood samples were obtained via cardiac puncture, and finally, pancreas tissue samples were extracted quickly and the rats were sacrificed. Plasma amylase (Scientific Research Special Hangzhou Eastbiopharm Co. Ltd. Hangzhou, China), IL-6 (Scientific Research Special Hangzhou Eastbiopharm Co. Ltd. Hangzhou, China), and IL-1beta (AssayPro, USA) levels were tested in accordance with the manufacturer’s instructions ([ELISA] kits).

In order to investigate pancreatic tissues histopathologically, the samples were fixed in 10% buffered formaldehyde and paraffin-embedded. Hematoxylin and eosin dying was performed and a photomicroscope examination was accomplished (Olympus BX 51; Tokyo, Japan) in line with the Schoenberg grading system.^13^

Histopathological evaluation was examined by a pathologist who was blind for the groups. The parameters evaluated were edema, inflammation, vacuolization, and necrosis. Pancreatic tissue specimens were examined by light microscopy as described by Schoenberg et al^13^ previously.

Pancreatic apoptosis was examined by terminal deoxynucleotidyl transferase-mediated nick-end-labeling (TUNEL) technique using ApopTag Plus Peroxidase, In Situ Apoptosis Detection Kit, Chemicon Int., Temecula, CA kit.

### Statistical Analysis

Evaluations for results are given as mean ± standard deviation. Comparisons of means between groups were evaluated using Student’s *t*-test or one-way analysis of variance for normally distributed data and Mann–Whitney *U* test or Kruskal–Wallis test for nonparametric data. *P* < .05 value was taken as a statistically significant value. The Statistical Package for Social Sciences version 20.0 software (IBM Corp.; Armonk, NY, USA) was used for statistical analysis.

## Results

**Figure 1.**
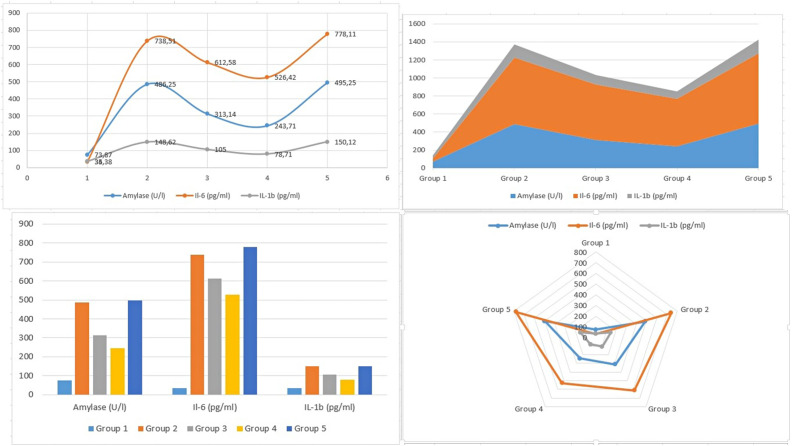
Results of the biochemical parameters of the study groups.

Serum amylase, IL-6, and IL-1beta levels are shown in Figure 1. When the biochemical results obtained for the groups are examined, they will be seen that it is as follows. From group 1 to group 5 for amylase (U/L); 73.87 ± 18.19; 486.25 ± 101; 313.14 ± 74.95; 243.71 ± 56.87; 495.25 ± 106.79 and for Il-6 (pg/mL); 34.38 ± 18.12; 738.51 ± 88.24; 612.58 ± 86.12; 526.42 ± 46.14; 778.11 ± 40.26 and for IL-1b (pg/mL); 35 ± 14.7; 148.62 ± 20.68; 105 ± 20.13; 78.71 ± 16.18; 150.12 ± 24.8 were detected, respectively.

**Figure 2.**
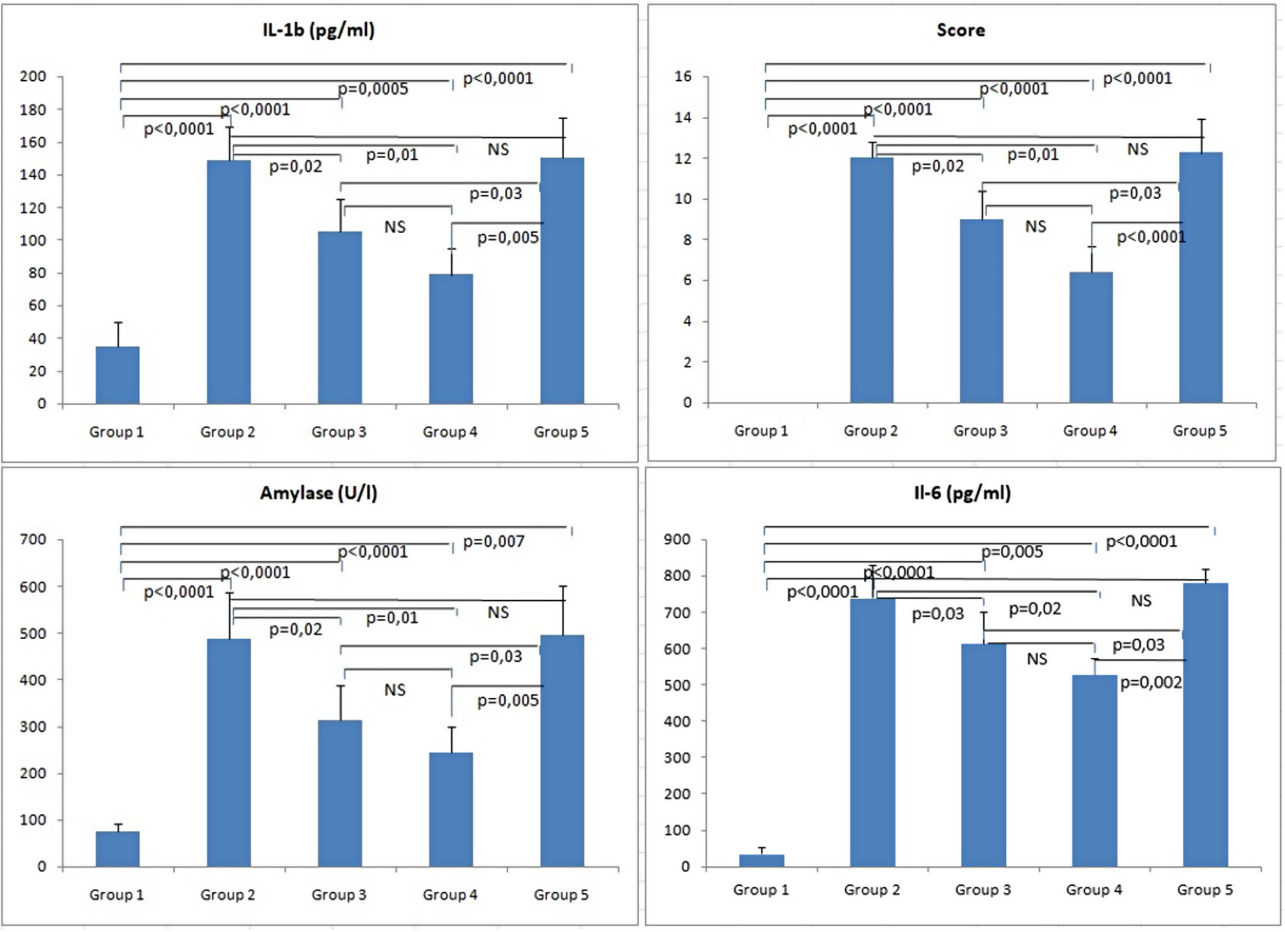
Bar graphics and *P* values of the biochemical parameters among study groups. Bonferroni, multiple comparisons. The mean difference is significant at the 0.05 level, and *P *< .0125 was considered statistically significant after Bonferroni’s correction. NS, not significant.

In the acute pancreatitis groups, serum amylase, IL-6, and IL-1b levels were statistically significantly raised compared to the sham group (*P *< .01). AP (group 2) and placebo (group 5) groups’ test results were similar in terms of serum biochemical parameters. Moreover, serum amylase, IL-6, and IL-1b levels statistically significantly diminished in the GLM administrated (groups 3 and 4) groups compared to the AP group (*P *< .05) (Figure 2).

### Pathological Evaluations

**Figure 3.**
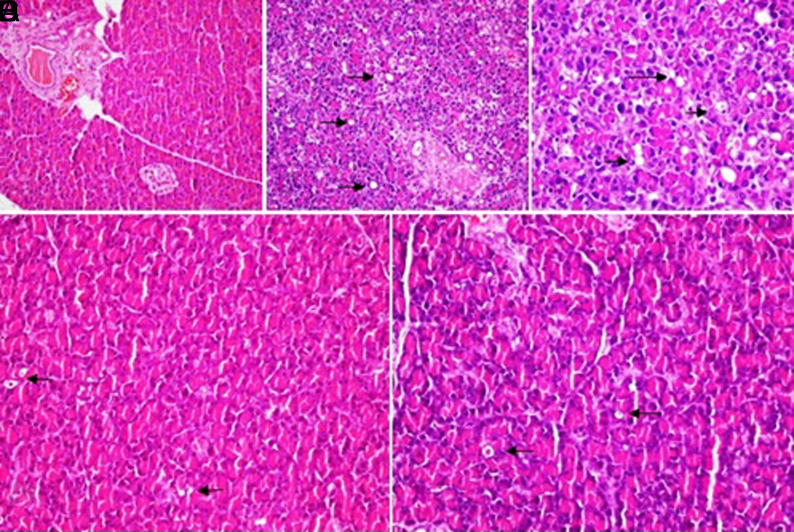
Hematoxylin and eosin staining of pancreatic tissue samples of all groups. (A) Sham group with normal pancreatic tissue appearance, 20× magnification. (B) Acute pancreatitis group, intralobular polymorphonuclear cell infiltration, vacuolation, necrosis, and edema are seen, 20× magnification. (C) Serum saline-treated placebo group, vacuolation, edema, and inflammation still remain, 40× magnification. (D) Golimumab low-dose treatment group, reduced polymorphonuclear cells, vacuolization, and edema with a low dose, 20× magnification. (E). Golimumab high-dose treatment group, 20× magnification. (Polymorphonuclear cell infiltration, vacuolation, necrosis, and edema are marked with black arrows).

**Figure 4.**
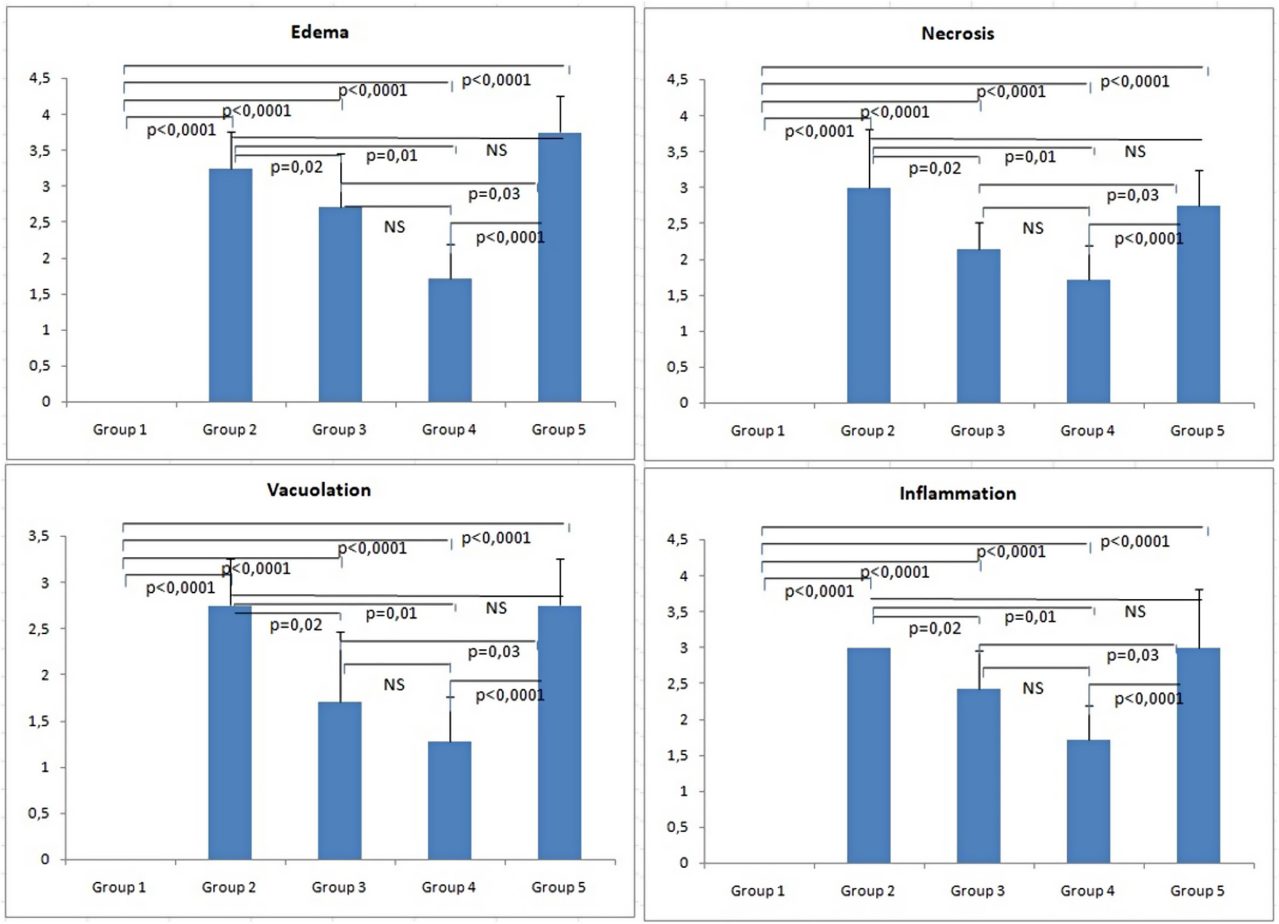
Bar graphics and *P* values of Schoenberg scores among study groups.

**Figure 5.**
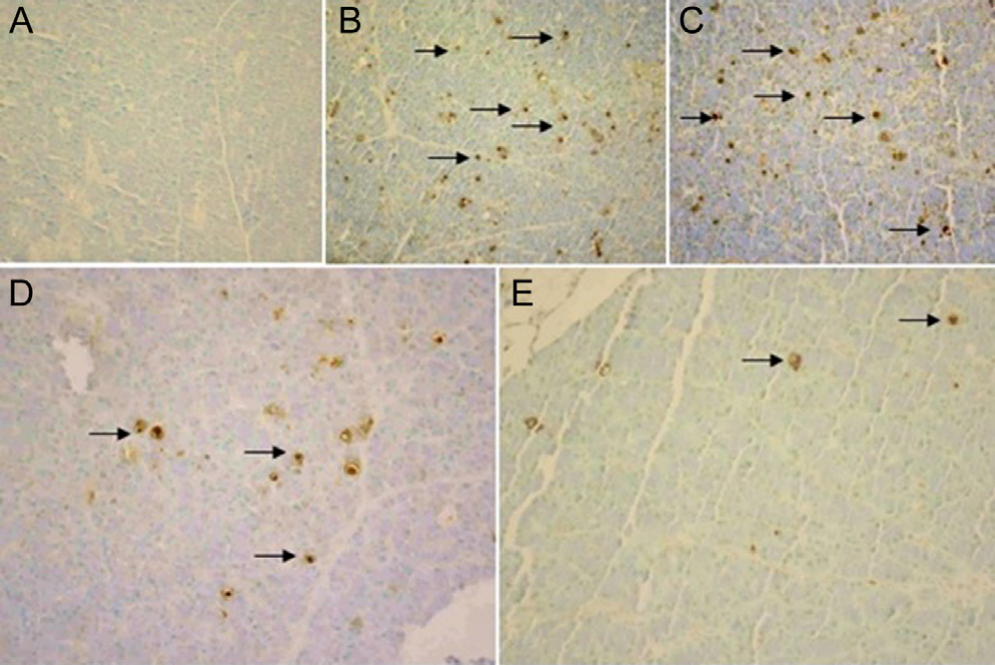
Apoptotic status of all study groups evaluated with transferase-mediated nick-end-labeling (TUNEL) technique. (A) Sham group. No apoptotic cell is seen. (B) Acute pancreatitis group. Increased apoptotic cells are seen. An apoptotic index is 4.7%. (C) Serum saline-treated placebo group, similar features with acute pancreatitis group. The apoptotic index is 5.3%. (D. Golimumab low-dose treatment group. Decreased apoptotic cells are seen with low doses. The apoptotic index is 1.7%. (E) Golimumab high-dose treatment group. The apoptotic index is 1.7%. (TUNEL staining, 20× magnification. Apoptotic cells are marked with black arrows).

**Figure 6.**
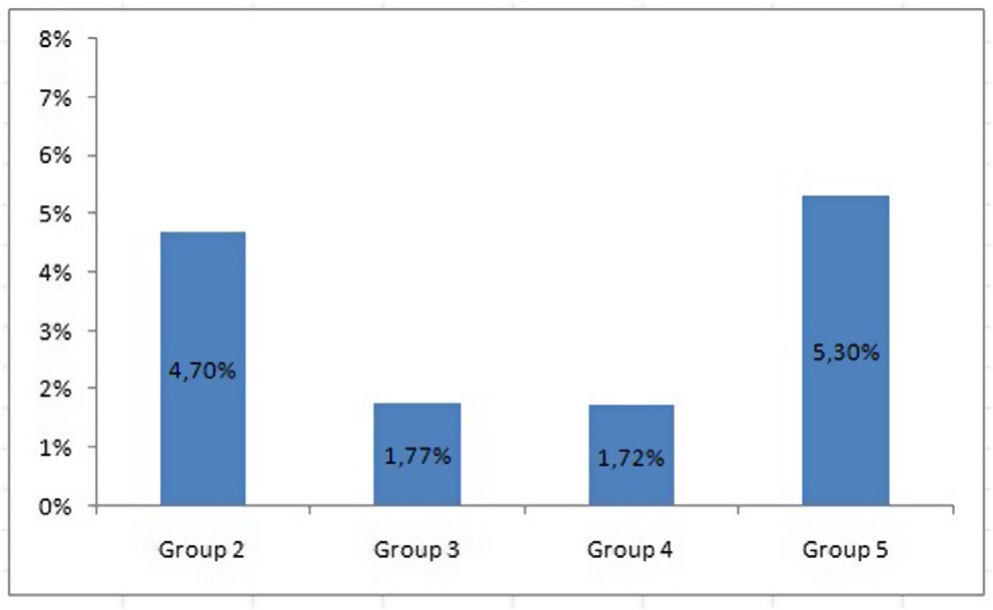
Bar graphic of apoptotic scores among study groups.

Histopathological evaluation, intense edema and inflammation, significant vacuolization, and fat necrosis were found in the acute pancreatitis group (Figure 3). Golimumab treatment was found to significantly reduce edema formation, inflammation, vacuolization, and fat necrosis of pancreatic tissues (*P *< .05, Table 1). The pathological scores of the placebo group were similar to the AP group. In the GLM treatment groups, the Schoenberg scores were significantly lower (*P *< .05) (Figure 4). In addition, GLM significantly reduced pancreatic apoptosis (Figures 5 and 6).

## Discussion

Acute pancreatitis is a life-threatening disease that can lead to life-threatening consequences. In AP, mortality rates are significantly increased, especially in the presence of systemic inflammatory response and multiple organ failure. The severe inflammatory response has been shown to play a pivotal role in the pathophysiology of AP.^14-19^

Despite the new treatment studies carried out in today’s conditions in the treatment of AP, it could not go beyond supportive treatment. When AP develops, proteolytic enzymes are activated due to the cytokines released.^1,2,4^ As a result of this enzyme activation, the volume in the microcirculation goes out of the vessel and causes erosion and even necrosis of the tissues. Although it causes serious volume loss, it causes serious circulatory failures that can cause organ failures. If the inflammation in the pancreas is continuous, it causes more release of toxic cytokines. As a result of intense cytokine release from monocytes and macrophages, the pancreas is irreversibly lost due to necrosis and tissue loss. As it is known, these cytokines: IL-1, IL-2, IL-6, IL-8, IL-10, TNF-a, and platelet-activating factor are the main known cytokines. In this sense, it is obvious that by partially preventing this cytokine discharge and stopping this activation, the AP complications that have developed will be prevented.^1,18,19^

When looking at the study of Sun et al^15^ on Behçet’s patients, it has been suggested that GLM may prevent disease-related complications by controlling inflammation. Thus GLM’s favorable anti-inflammatory effect was a beginning point for our research. In our current experimental study, GLM both decreased pancreatic inflammation and apoptosis which is a target of AP treatment. Also, GLM improved biochemical results in this current research which may be another encouraging outcome.

When the available literature is reviewed, some authors pointed out the probable links between diabetes and the likelihood of pancreatitis development. Regardless of the etiology of pancreatitis, both acute and chronic pancreatitis pathogenesis may be associated with oxidative stress and inflammation.^20-23^ In the study conducted by Quattrin et al^21^ it was suggested that endogenous insulin secretion increased and exogenous insulin requirement decreased after GLM treatment in type 1 diabetes patients. This study clearly showed that after AP development, GLM treatment both suppresses inflammation in the pancreas and reduces acinar and beta-cell loss by preventing apoptosis. In another study conducted with children with Type 1 diabetes, the use of another TNF-α-blocking drug etanercept showed similar favorable effects on the pancreas.^22^ Thus, these findings may support why GLM may be a favorable therapeutic in case of AP and AP-related pancreatic complications.

In the previous study conducted by Kaplan et al^23^ anakinra treatment, which is an IL-1 receptor antibody, showed beneficial biochemical and histopathological effects on the experimental rat AP model. In that study, strong suppression of inflammation by anakinra protected the pancreas as with high-dose GLM treatment groups as in this current study.^23^ In another experimental AP rat model accomplished by Tanoglu et al. the use of trimetazidine had been found to significantly reduce inflammation in AP and prevented pancreatitis-associated complications. Similarly, in this current experimental study, GLM significantly reduced levels of inflammatory cytokines in AP treatment groups and clearly suppressed pancreatic inflammation.^24^ Especially favorable positive GLM effects on pancreatic tissue may support its candidate therapeutic agent in AP cases.

In a study by Nemoto et al^25^ they evaluated the effectiveness and long-term use of GLM. Regarding the long-term use and dosage of GLM in daily practice for rheumatoid arthritis (RA) cases, it was seen that the symptoms of RA were improved with increasing GLM dose in the long-term treatment course. Similarly, in our study, it was found that inflammation was suppressed better, apoptosis was significantly reduced with high dose (10 mg/kg) GLM treatment when compared with the low-dose GLM group. Thus, in line with our current results, it can be speculated that relatively high doses of GLM are more effective and also safe in the treatment of AP.

In conclusion, our current study showed that GLM is an effective and safe drug in the treatment of AP. Moreover, it can be suggested that GLM will be a favorable therapeutic option in acute AP. Due to the presence of limited treatment options for AP, our current data seem promising in terms of decreasing inflammation and preventing pancreatic complications.

## Figures and Tables

**Table 1.** Schoenberg scores of the study groups

**Groups**	**Edema**	**Inflammation**	**Vacuolation**	**Necrosis**	**Score**
Group 1 (sham)	0	0	0	0	0
Group 2 (pancreatitis)	3.25 ± 0.5	3	2.75 ± 0.5	3 ± 0.81	12 ± 0.81
Group 3 (golimumab 5 mg/kg)	2.71 ± 0.75	2.42 ± 0.53	1.71 ± 0.75	2.14 ± 0.37	9 ± 1.41
Group 4 (golimumab 10 mg/kg)	1.71 ± 0.48	1.71 ± 0.48	1.28 ± 0.48	1.71 ± 0.48	6.42 ± 1.27
Group 5 (placebo)	3.75 ± 0.5	3 ± 0.81	2.75 ± 0.5	2.75 ± 0.5	12.25 ± 1.70
